# Supplementary material for: Diagnostic Tool for the Identification of Bactrocera dorsalis (Hendel) (Diptera: Tephritidae) Using Real-Time PCR
Source: Insects. 2024 Jan 8;15(1):44. doi: 10.3390/insects15010044 (PMC10815988; doi:10.3390/insects15010044)
Supplement: Supplementary file 1 [file insects-15-00044-s001.zip › insects-2752703-supplementary.pdf]

Supplementary Materials

Development of a diagnostic tool for the early and reproducible identification of *Bactrocera dorsalis* (Hendel) (Diptera Tephritidae) using Real-Time PCR with TaqMan probe technology

Rizzo Domenico[1], Zubieta Claudia Gabriela[1], Sacchetti Patrizia[2], Andrea Marrucci[1,4], Miele Fortuna[3], Ascolese Roberta[3,5], Nugnes Francesco[3], Bernardo Umberto[3,*]

[1] Laboratory of Phytopathological Diagnostics and Molecular Biology, Plant Protection Service of Tuscany, Via Ciliegiole 99, 51100 Pistoia, Italy; domenico.rizzo@regione.toscana.it; claudiagabriela.zubieta@regione.toscana.it; andrea.marrucci93@gmail.com.
[2] University of Florence, Department of Agriculture, Food, Environment and Forestry (DAGRI), Piazzale delle Cascine, 18, 50144 Florence (Italy); patrizia.sacchetti@unifi.it.
[3] Institute for Sustainable Plant Protection (IPSP) – CNR, P.le Enrico Fermi 1, 80055 Portici, Italy; roberta.ascolese@ipsp.cnr.it; fortuna.miele@ipsp.cnr.it; francesco.nugnes@ipsp.cnr.it; umberto.bernardo@ipsp.cnr.it.
[4] Department of Agricultural Food and Agro-Environmental Sciences, University of Pisa, Via del Borghetto 80, I-56124 Pisa (Italy); andrea.marrucci93@gmail.com.
[5] Department of Biology, University of Naples Federico II, Via Vicinale Cupa Cintia 21, 80126 Napoli (Italy); roberta.ascolese@unina.it.
* Correspondence: umberto.bernardo@ipsp.cnr.it; Tel.: +3906499327823

Figure S1. Inclusiveness of the qPCR probe assay. Eighteen sequences from different populations of B. dorsalis, geographically distant, were retrieved from GenBank through alignments based on the in-silico theoretical qPCR probe amplicon.

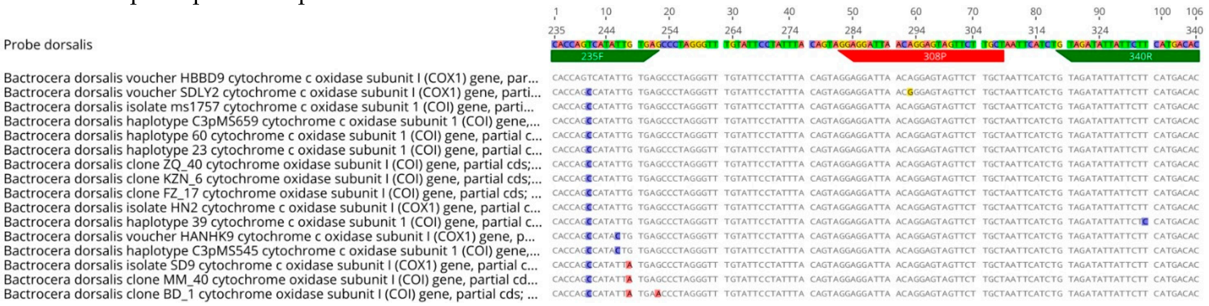

Figure S2. Alignments resulting from the in silico theoretical Probe amplicon and sequences of the related organisms present in GenBank. More than 200 different *Bactrocera* specimens present and detected in different source areas were compared through the sequence alignments in the network.

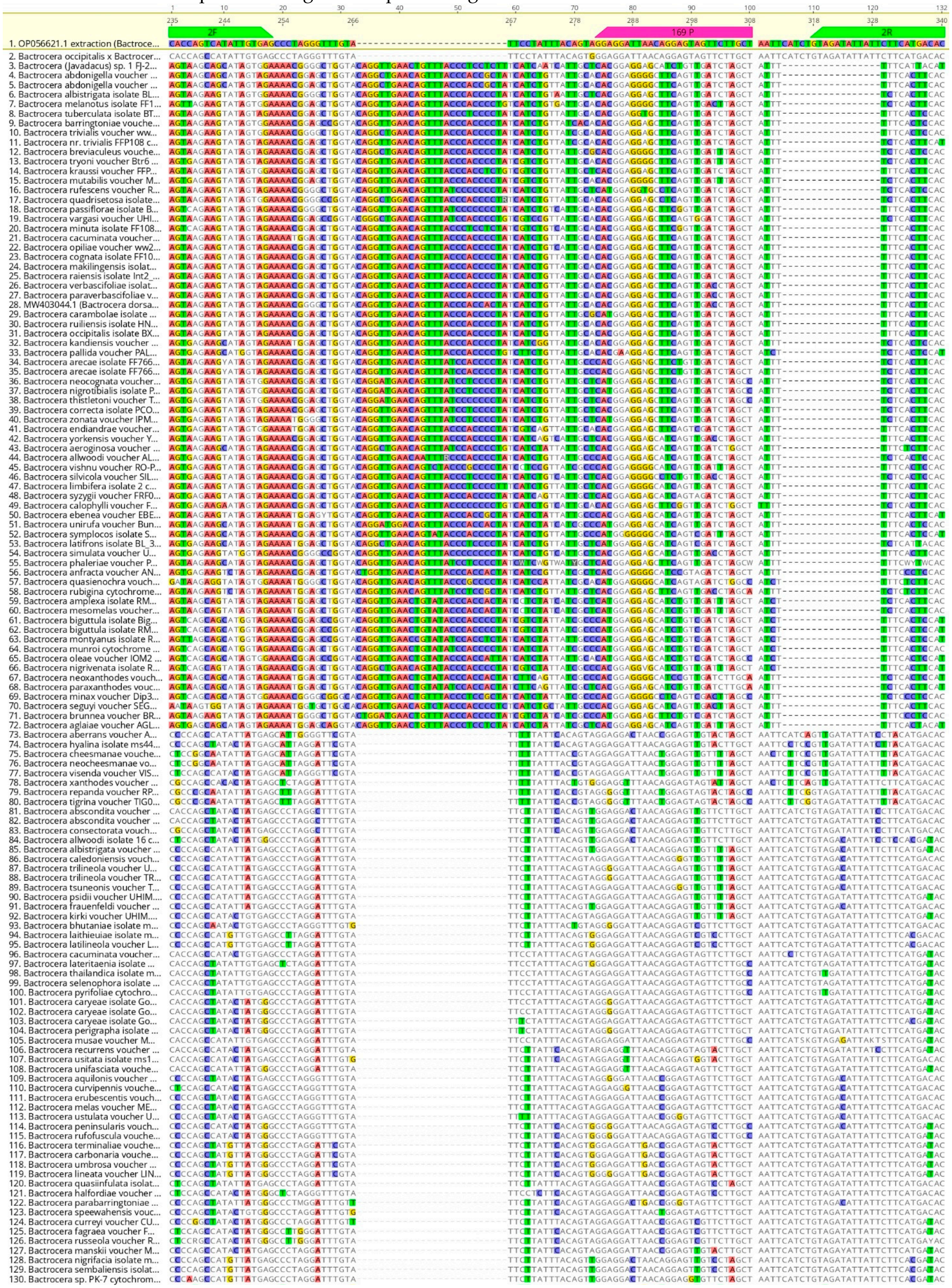

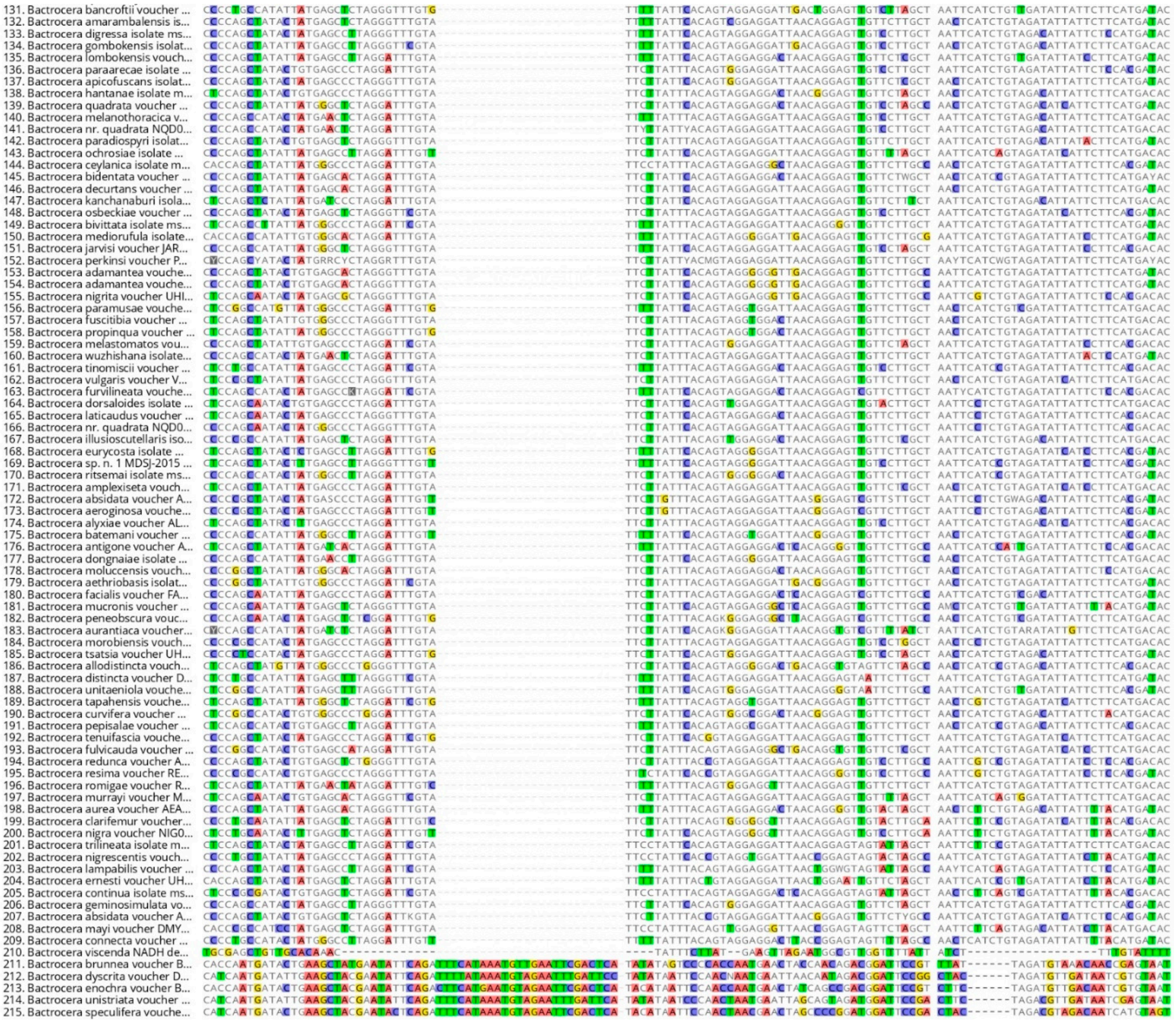

Table S1. Target and non-target list with indication of Cqs detected in single gene amplification reactions with the qPCR probe

Species	Sample code	Concentration	Cq
Anastrepha fraterculus (Wiedemann, 1830)	MR 000752	10 ng/µL	n/a
	MR 000795	10 ng/µL	n/a
Anastrepha leptozona Hendel 1914	MR 000817	10 ng/µL	n/a
	MR 000823	10 ng/µL	n/a
	MR 001658	10 ng/µL	n/a
Anastrepha ludens (Loew, 1873)	MR 000753	10 ng/µL	n/a
	MR 000820	10 ng/µL	n/a
	MR 001659	10 ng/µL	n/a
	MR 001710	10 ng/µL	n/a
	MR 001721	10 ng/µL	n/a
Anastrepha obliqua (Macquart, 1835)	MR 000821	10 ng/µL	n/a
	MR 001715	10 ng/µL	n/a
Anastrepha serpentina (Wiedemann, 1830)	MR 000283	10 ng/µL	n/a
	MR 000822	10 ng/µL	n/a

	MR 001701	10 ng/μL	n/a
	MR 001689	10 ng/μL	n/a
	MR 001722	10 ng/μL	n/a
Bactrocera dorsalis (Hendel, 1912)	MR 001712	10 ng/μL	22.78
	MR 001720	10 ng/μL	21.36
	MR 001709	10 ng/μL	21.40
	MR 001718	10 ng/μL	21.26
	MR 001716	10 ng/μL	21.16
	MR 000814	10 ng/μL	21.12
	MR 001563	10 ng/μL	20.98
	MR 001713	10 ng/μL	21.10
	MR 001638	10 ng/μL	20.87
	MR 001714	10 ng/μL	21.45
	MR 001717	10 ng/μL	21.08
	MR 001711	10 ng/μL	17.56
	MR 001719	10 ng/μL	16.52
	MR 000801	10 ng/μL	17.24
	MR 001683	10 ng/μL	16.87
	MR 001684	10 ng/μL	17.45
	MR 000239	10 ng/μL	17.25
	MR 001697	10 ng/μL	16.98
	MR 001747	10 ng/μL	17.28
	MR 000764	10 ng/μL	17.19
Bactrocera latifrons (Hendel, 1915)	MR 000752	10 ng/μL	n/a
	MR 000823	10 ng/μL	n/a
Bactrocera oleae (Rossi, 1790)	MR 001658	10 ng/μL	n/a
Bactrocera zonata (Saunders, 1842)	MR 000753	10 ng/μL	n/a
Ceratitis capitata (Wiedemann, 1824)	MR 000820	10 ng/μL	n/a
	MR 000821	10 ng/μL	n/a
	MR 000283	10 ng/μL	n/a
	MR 000822	10 ng/μL	n/a
Cydia pomonella (Linnaeus, 1758)	MR 000259	10 ng/μL	n/a
	MR 000790	10 ng/μL	n/a
Grapholita molesta (Busck, 1916)	MR 001617	10 ng/μL	n/a
Rhagoletis cerasi(Linnaeus, 1758)	MR 001618	10 ng/μL	n/a
Rhagoletis completa(Cresson, 1929)	MR 001648	10 ng/μL	n/a
	MR 001619	10 ng/μL	n/a
Thaumatotibia leucotreta(Meyrick, 1913)	MR 001678	10 ng/μL	n/a
	MR 001620	10 ng/μL	n/a
	MR 001621	10 ng/μL	n/a
